# Hypermagnesemia in a 20-month-old healthy girl caused by the use of a laxative: a case report

**DOI:** 10.1186/s13256-021-02686-9

**Published:** 2021-03-10

**Authors:** Kotaro Araki, Yuhei Kawashima, Miyuki Magota, Norio Shishida

**Affiliations:** Department of Pediatrics, Okinawa Prefectural Yaeyama Hospital, 584-1 Maezato , Ishigaki-shi, Okinawa 907-0002 Japan

**Keywords:** Hypermagnesemia, Constipation, Laxative, Healthy child

## Abstract

**Background:**

Hypermagnesemia can be a fatal condition and should be diagnosed early on. Most reports of hypermagnesemia have been of adults with impaired renal function. We describe the case of a pediatric patient without renal dysfunction who developed severe hypermagnesemia.

**Case presentation:**

A healthy 20-month-old Asian girl presented to our emergency department with episodes of vomiting and a reduced level of consciousness. The neurological examination showed a symmetric decrease in muscle tone, and the deep tendon reflexes were decreased. On admission, her magnesium (Mg) level was 11.0 mg/dL after receiving magnesium oxide for 4 days because of constipation. She was immediately administered calcium gluconate infusion (3.9 mEq), and then was continuously infused with it (0.23 mEq/h) as a Mg antagonist to cardiac side effects. She was kept hydrated with 0.9% sodium chloride to maintain good urine output to excrete the Mg. The level of the serum Mg decreased to 2.4 mg/dL, enabling her to regain consciousness. During 5 years of follow-up, she was neurologically well, without the recurrence of hypermagnesemia.

**Conclusions:**

Even in the absence of significant renal dysfunction, the prescription of a laxative containing Mg for constipation can result in severe hypermagnesemia. In addition, the symptoms of hypermagnesemia are nonspecific, and early diagnosis is difficult unless it is actively suspected.

## Background

Hypermagnesemia is a rare condition that is usually iatrogenic and may be caused by intravenous administration of magnesium (Mg) or oral ingestion of antacids or laxatives containing Mg [1, 2]. The level of serum Mg is regulated mainly through gastrointestinal absorption and renal excretion [3]. The kidney is the most important organ involved in the regulation of Mg. Although hypermagnesemia is relatively rare among pediatric patients, it commonly occurs in older people with renal failure. However, rare pediatric cases have been reported to show symptomatic hypermagnesemia in several situations such as excessive use of laxatives or a surgical procedure [4, 5]. The symptoms are nonspecific and often difficult to diagnose at an early stage, which can be fatal. We present a case of a pediatric patient with no medical history who developed hypermagnesemia after ingestion of a normal dosage of magnesium oxide (MgO) as a laxative.

## Case presentation

A 20-month-old healthy Asian girl weighing 10.3 kg without any medical history, in a coma, and with progressive generalized weakness was admitted to our emergency department. She had complained of constipation, and 200 mg of MgO in divided doses daily had been prescribed earlier for 4 days by her family physician. According to the family, there was no stool discharge for 5 days, and the patient had episodes of vomiting twice in the morning in addition to a reduced level of consciousness. No remarkable findings were observed regarding her birth history, growth rate, or family history. She was not taking any medication, except for MgO as a laxative.

The vital signs on admission were as follows: temperature of 97.1 °F (36.2 C°), heart rate of 104 beats per minute, systolic and diastolic pressures of 110 and 60 mmHg, respectively, respiratory rate of 28 breaths per minute, and oxygen saturation of 98% in room air. The physical examination revealed that the state of consciousness was E2V1M1 on the Glasgow Coma Scale. Her extremities did not respond to pain stimulus; however, her facial expression was set and showed little display of feeling. The neurological examination showed a symmetric decrease in muscle tone. The deep tendon reflexes were also decreased, and the pupillary reflexes were sluggish. No abnormalities were observed in her respiratory status or heart sounds. The abdomen was distended with diminished bowel sounds, and a mass of stool was palpable.

The laboratory findings showed that the patient had fatal hypermagnesemia (11.0 mg/dL) and hyperglycemia (377 mg/dL). The hemogram showed leukocytosis (white blood cell count 26,760/μL) with bands accounting for 82.6% and no anemia (hemoglobin 13.9 mg/mL). Other results included the following: serum calcium, 9.3 mg/dL; serum sodium, 130 mEq/L; serum potassium, 4.0 mEq/L; serum chloride, 100 mEq/L; serum creatine, 0.17 mg/dL; blood urine nitrogen, 30.4 mg/dL; total bilirubin, 0.2 mg/dL; venous blood pH, 7.33; partial pressure of carbon dioxide (PaCO_2_), 36.5 mmHg; and partial pressure of oxygen (PO_2_), 60.6 mmHg. An electrocardiogram showed normal and no block patterns. The blood cultures were negative. The patient was diagnosed with fatal hypermagnesemia caused by intoxication from a laxative containing MgO. She was instantly administered calcium gluconate infusion at 3.9 mEq for over 10 min. She was then continuously infused with calcium gluconate at 0.23 mEq/hour as a Mg antagonist to reverse the neuromuscular and cardiac effects of hypermagnesemia. She was kept hydrated with 0.9% sodium chloride to maintain good urine output to excrete the Mg, and a total of 720 mL was administered over the next 12 hours. An enema was performed, which expelled a large mass of stool and a large amount of muddy stool. After 2 hours, her serum Mg level was decreased to 5.9 mg/dL, and she regained consciousness to a level of being verbally responsive. Over the next 12 hours, her neurologic status continued to improve. Her serum Mg and glucose levels decreased to 2.4 mg/dL and 116 mg/dL, respectively. The patient recovered, and was discharged without recurrence of hypermagnesemia. At the follow-up visit 3 days later, her serum Mg level was 1.9 mg/dL. Five years after discharge, during a follow-up evaluation, she was neurologically well and had returned to her baseline status without a recurrence of hypermagnesemia and hyperglycemia.

## Discussion and conclusions

This is the first report on hypermagnesemia caused by normal dosage of a laxative in a healthy child with no medical history. Most reports of hypermagnesemia have been of adults with impaired renal function [6, 7], and there have been few reports of hypermagnesemia in children. There has been only one reported case of hypermagnesemia with normal renal function caused by the use of a laxative in children. However, the patient had a medical history of sacral agenesis, had undergone surgery for hydronephrosis secondary to ureterovesical obstruction, and had ingested an excessive amount of laxative orally [4].

Mg is an important and the second most common intracellular divalent cation after calcium, and is an essential mineral for all cells and normal biological function. Mg administered orally is regulated by absorption mainly through the small intestine, although it is sometimes taken up via the large intestine and excreted through the kidneys. Its absorption rate varies widely (ranging from 24 to 75%), and the rest is excreted in feces [3, 8]. Intestinal absorption is mainly dependent on the status of the serum Mg. When the Mg level is high, a lower amount of Mg is absorbed in the gut. Therefore, absorption of Mg is relatively low when its intake is high. Renal Mg excretion is very efficient because the thick ascending limb of Henle has the capacity to reject the reabsorption of Mg in the case of hypermagnesemia [8].

Although serum Mg concentrations rarely rise above the maximum limit, administration of Mg may result in fatal hypermagnesemia. Many cases of hypermagnesemia have been reported, even in the presence of normal renal function. The absorption of orally administered Mg is regulated; however, the rate of absorption from the gastrointestinal mucosa has been reported to increase in the presence of gastrointestinal lesions such as ulcers, bleeding, and inflammation [9]. In addition, when intestinal movement is weakened by paralytic ileus, the administered Mg stagnates in the gastrointestinal tract for a prolonged period. Consequently, the absorption rate of Mg increases. Further studies have reported that hypermagnesemia itself causes stagnation of intestinal peristalsis, and contributes to ileus [10]. Our patient had no instances of gastrointestinal bleeding; nonetheless, persistent constipation was present. We realized that the lack of disimpaction of the fecal blockage prior to Mg administration (which caused ileus) was the cause of the hypermagnesemia.

Symptoms of hypermagnesemia are characterized by disorders of the neuromuscular, respiratory, and cardiac functions, and the symptoms that appear depend on the serum Mg concentration. The systemic manifestations related to dose and serum levels of Mg are summarized in Table 1 [11]. Because these symptoms are nonspecific, early diagnosis is difficult unless the disease is proactively suspected. Whang *et al*. measured the serum Mg levels in 1033 blood samples from hospitalized patients [12]. Only 13% (7/59) of the hypermagnesemia patients were identified through physician-initiated requests. The authors noted that the majority of the hypermagnesemia cases were not suspected early at the onset. In our case, the serum Mg level was 11.0 mg/dL. This was consistent with the disappearance of the deep tendon reflex. These values were noted just before the appearance of the cardiac block, and would have been fatal if the diagnosis had been delayed.

**Table 1:** Manifestations of hypermagnesemia in relation to serum magnesium levels

Serum Mg level (mg/dL)	Clinical manifestations
1.7–2.4	Normal serum level
5–8	Nausea, vomiting, cutaneous, flushing, hypotension, bradycardia
9–12	Absence of deep tendon reflexes, somnolence
>15	Respiratory depression, paralysis, complete heart block
>20	Cardiac arrest in the asystole

In conclusion, we report a case of a healthy pediatric patient who did not have renal dysfunction; nevertheless, she had developed severe hypermagnesemia after oral ingestion of a laxative containing Mg. We posit that severe hypermagnesemia can occur in the absence of preexisting renal dysfunction, particularly if the patient has fecal impaction.

## Data Availability

Not applicable.

## Abbreviations

Mg: Magnesium
MgO: Magnesium oxide
